# Vitamin B6 *Via* p-JNK/Nrf-2/NF-κB Signaling Ameliorates Cadmium Chloride-Induced Oxidative Stress Mediated Memory Deficits in Mice Hippocampus

**DOI:** 10.2174/1570159X22999240730154422

**Published:** 2024-07-31

**Authors:** Abdul Nasir, Mujeeb Ur Rahman, Manzar Khan, Muhammad Zahid, Muhammad Shahab, Hongjun Jiao, Amir Zeb, Shahid Ali Shah, Haroon Khan

**Affiliations:** 1 Medical Research Center, Second Affiliated Hospital of Zhengzhou University, Zhengzhou, Henan, China;; 2 Department of Zoology, Islamia College Peshawar, Khyber Pakhtunkhwa, Pakistan;; 3 Department of Zoology, Hazara University Mansehra, Khyber Pakhtunkhwa, Pakistan;; 4 State Key Laboratories of Chemical Resources Engineering, Beijing University of Chemical Technology, Beijing 100029, PR China;; 5 Department of Pharmacy, Second Affiliated Hospital of Zhengzhou University, Zhengzhou, Henan, China;; 6 Department of Natural and Basic Sciences, University of Turbat, Turbat 92600, Pakistan;; 7 Department of Biology, University of Haripur, Khyber Pakhtunkhwa, Pakistan;; 8 Department of Pharmacy, Abdul Wali Khan University, Mardan, Khyber Pakhtunkhwa, Pakistan

**Keywords:** Oxidative stress, neurotoxicity, neurodegenerative disease, Alzheimer's disease, neuroinflammation, anti-inflammatory

## Abstract

**Background:**

Cadmium chloride (Cd) is a pervasive environmental heavy metal pollutant linked to mitochondrial dysfunction, memory loss, and genetic disorders, particularly in the context of neurodegenerative diseases like Alzheimer's disease (AD).

**Methods:**

This study investigated the neurotherapeutic potential of vitamin B6 (Vit. B6) in mitigating Cd-induced oxidative stress and neuroinflammation-mediated synaptic and memory dysfunction. Adult albino mice were divided into four groups: Control (saline-treated), Cd-treated, Cd+Vit. B6-treated, and Vit. B6 alone-treated. Cd and Vit. B6 were administered intraperitoneally, and behavioral tests (Morris Water Maze, Y-Maze) were conducted. Subsequently, western blotting, antioxidant assays, blood glucose, and hyperlipidemia assessments were performed.

**Results:**

Cd-treated mice exhibited impaired cognitive function, while Cd+Vit. B6-treated mice showed significant improvement. Cd-induced neurotoxic effects, including oxidative stress and neuroinflammation, were observed, along with disruptions in synaptic proteins (SYP and PSD95) and activation of p-JNK. Vit. B6 administration mitigated these effects, restoring synaptic and memory deficits. Molecular docking and MD simulation studies confirmed Vit. B6's inhibitory effect on IL-1β, NRF2, and p-JNK proteins.

**Conclusion:**

These results highlight Vit. B6 as a safe therapeutic supplement to mitigate neurodegenerative disorders, emphasizing the importance of assessing nutritional interventions for combating environmental neurotoxicity in the interest of public health.

## 1 INTRODUCTION

Neurodegenerative disease (ND) encompasses a gradual loss of neurons in the brain or peripheral nervous system and is often linked to protein aggregation, immunological dysregulation, and metabolic challenges [1, 2]. Neurological disorders have a significant global impact, encompassing a wide range of conditions such as epilepsy, Alzheimer's disease, stroke, and headaches [3]. Recent reports highlight the staggering statistic that these disorders collectively affect approximately one billion individuals (15% of the global population). Among these conditions, ND encompasses a range of manifestations, such as brain injuries, neuroinfections, multiple sclerosis, and Parkinson's disease [4]. The pathophysiological hallmark of ND has been attributed to a complex interplay of genetic factors (70%) and environmental influences (30%) [5]. In environmental factors, air pollution containing toxins and heavy metals, such as Cadmium Chloride (Cd), has emerged as a potential contributor to ND, including Alzheimer's disease (AD) and Parkinson's disease (PD) [6]. AD, characterized by cognitive and memory decline along with behavioral, physical, and linguistic challenges, remains a significant neurodegenerative condition [7-9]. Key contributors to AD pathogenesis include oxidative stress, mitochondrial dysfunctions, amyloid-beta (Aβ) deposition, tau protein phosphorylation, and neural network dysfunction [10]. The oxidative stress-induced imbalance between oxidants and antioxidants further exacerbates AD progression, leading to neuronal damage and apoptosis [11, 12]. Moreover, oxidative stress suppresses the endogenous antioxidant defense system, including Nuclear factor erythroid 2-related factor 2 (NRF2), Novel Heme Oxygenase-1 (HO-1), and triggers oxidative markers such as Nuclear Factor Kappa B (Nfkβ) and Toll-Like Receptor TLR4, leading to neuroinflammation, synaptotoxicity and memory impairment [13, 14]. Memory impairment, a common feature of various dementias, including AD and PD, further underscores the urgency of understanding the underlying mechanisms and developing effective interventions [15, 16].

Cd is characterized as a toxic heavy metal owing to its harmful effect on cellular and metabolic systems in both humans and animals, posing a significant threat [17]. Cd salts are recognized as the most hazardous environmental compounds, persistently endangering human health due to their resistance to decomposition and subsequent presence in the environment. Human exposure to Cd occurs mainly through food chain contamination. Cd exposure primarily occurs through consuming contaminated foods, and prolonged exposure to Cd induces toxic effects, including neurological disturbances and changes in normal brain neurochemistry [18, 19]. In search of effective and safe countermeasures against Cd-induced oxidative stress and inflammation, exploration of therapeutic agents remains a critical pursuit [20, 21]. Emerging approaches to address these challenges involve novel agents, such as different nutrients and bioactive compounds. Among these, Vitamin B6 (Vit. B6) holds a well-known reputation for its antioxidant properties and capacity to mitigate neuroinflammation [22]. Vit. B6 is directly involved in memory impairment meditation, drawing attention to its potential to positively influence mild cognition, mitigate neurochemical alterations, and alleviate long-term cognitive deficits [23, 24].

In the current study, we explored the neuroprotective potential of Vit. B_6_ against Cd-induced AD-like neuropathology in adult mice. Building on previous studies demonstrating Vit. B_6_ neuroprotective properties both *in vivo* and *in vitro* [25, 26], our aim is to evaluate its antioxidants and anti-inflammatory effects in the context of AD-associated neuropathological changes. By exploring the intricate interplay between Vit. B6, oxidative stress, and neuroinflammation in the presence of Cd, we strive to provide valuable insights into potential therapeutic strategies for managing neurodegenerative disorders.

## 2 MATERIALS AND METHODS

### 2.1 Chemicals

Cadmium chloride (Cat No. 655198), Vit. B_6_ (Cat No. 65-23-6), Phosphate Buffer Saline tablets (PBS), Sodium Dodecyl Sulphate (SDS), Ammonium per Sulphate (APS), acrylamide, biscrylamide, tris base, sodium chloride, potassium chloride, methanol and glycine were purchased from Sigma Chemical Co. (St. Louis, Mo, USA) and Dae-Jung Chemicals and Metals Co. Ltd. (Gyeonggi-do, Shiheung, South Korea).

### 2.2 Mice Grouping

In this study, adult albino mice (8-12 weeks old, n = 10) were purchased from the Veterinary Research Institute (VRI) Peshawar and carefully brought to the Neuro Molecular Medicine Research Center (NMMRC). Upon arrival, the Mice were individually housed in cages (Bio Base, China) and randomly assigned to one of the following groups: Control Group, mice treated with CdCl_2_ at 1 mg/kg, mice treated with CdCl_2_ (1 mg/kg) + Vit. B_6_ (300 µg/kg), mice treated with Vit. B_6_ (300 µg/kg). The mice (body weight 29 ± 3.1 g) were kept under a 12/12 light-dark cycle at a temperature of 25 ± 1.2°C. Water and food pellets are available *ad libitum*. The Cd (1 mg/kg) and Vit. B_6_ (300 µg/kg) were dissolved in saline and administrated intraperitoneal injections.

### 2.3 Behavior Tests

After completion of dosing, the effect of Vit. B6 on neuroinflammation and memory dysfunction-induced Cd, Y-maze and Morris Water Maze behavior tests were performed. The researcher carrying out the behavioral tests was kept blind to the tags and the mice in the treatment group.

#### 2.3.1 Morris Water Maze (MWM)

The MWM test was used to examine hippocampus-dependent long-term spatial learning abilities, as reported recently [27]. Morris water maze is a circular tank (100×40 cm) filled with water (26 cm depth) and has a temperature of 23°C. The Mice were trained twice daily for three days to become familiar with the submerged platform. In case the mice could not find a submerged platform, the failed mice sat on the platform for 10 seconds to memorize the platform. Twice a day of training, the mice were allowed to search the platform in the MWM within 60 sec. After five consecutive days of data collection, every test has data for the experimental groups. Following a two-day period of rest, the mice were subjected to a probing test in order to locate a concealed platform. In the probe test, the submerged platform was removed, and the mice were allowed to locate the submerged quadrant. The duration of time spent by the mice in the target quadrant was subsequently recorded.

#### 2.3.2 Y-Maze

The Y-maze test was performed according to the previously published procedure [28]. The apparatus has three arms of 50×10×20 (cm^3^ L×W×H) at an angle of 120^0^. The mice were trained to adjust to a new environment for 10 min every three days. The mice were allowed to explore the maze for 8 min in center-facing arm A. The mouse's entrance was noted each time, and the successive number of triplets was counted on the page. The output data determined the memory status of the mice.


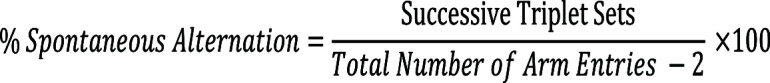


### 2.4 Western Blotting

All mice were sacrificed carefully after treatment and behavior investigation, and the whole brain was carefully collected. The hippocampus was separated in the test tube, and a 1:1 RNA ladder was added and stored at -4°C. Tissue Protein Extraction Reagent (T-PER) was added and homogenized for 3 min (Thermo Scientific). The supernatant was centrifuged for 25 mints at 14000 rpm and 3°C. A solution for the Bio-Rad protein assay was prepared to facilitate protein spectrometry analysis, measuring protein concentration at an absorbance of 595 nm. A 30-µg protein sample was ready for 15% SDS gel electrophoresis. The protein was transferred from gel to polyvinylidene difluoride (PVDF) membrane (Bio-Rad) through trans-blot (Bio-Rad). The primary antibodies were applied to the PVDF membrane for 12 hrs at 4°C. The antibodies were derived from mice. The secondary antibody was deployed for 4 hrs at room temperature. Later, the PVDF membrane results were developed on X-ray film using a chemiluminescence assay reagent.

### 2.5 Antioxidant Assay

#### 2.5.1 Lipid Peroxidation Determination (LPO) in Tissue

The LPO assay was carried out by employing a previously used protocol [29]. A polytron homogenizer was used to homogenize mouse brain tissues at 20 mM Tris-HCl, pH 7.4 (10 ml) at 4°C. The supernatant was obtained after centrifuging the homogenate at 100 g for 10 min at 4°C. For lipid peroxidation, freshly formed ferric or ferrous ammonium sulfate was added to brain homogenate tissues (40 ml) and incubated at 37°C for 30 min. Following that, 75 ml of 2-thiobarbituric acid (TBA 0.8%) solution was administered, which was made by dissolving TBA (400 mg) in distilled water (50 ml). A plate reader was used to read absorbance at 532 nm.

#### 2.5.2 Reduced Glutathione (GSH) Activity

The glutathione (GSH) level was detected, similar to the previously employed protocol, with slight modifications [30]. We mixed 0.2 mL of tissue supernatant with 2 mL of DTNB, then added 0.2 M phosphate buffer to make a final amount of 3 mL. After 10 min, the absorbance was read at 412 nm with a spectrophotometer with phosphate buffer and 5,5′-dithiobis-2-nitrobenzoic acid (DTNB) solution as blank and control, respectively. The final GSH activity was measured in mol/mg of protein.

#### 2.5.3 Glutathione-S-Transferase (GST)

Previously documented assay protocols were employed to measure GST levels [31]. We used freshly generated 1 mM 1-chloro-2,4-dinitrobenzene (CDNB) and 5 mM GSH solutions in 0.1 M phosphate buffer to determine GST activity. Three samples of the 1.2 mL reaction mixture were preserved in glass vials, and each of these mixes received 60 µL of homogenized tissue. Instead of tissue lysate, the blank contained water. Following that, 10 mL aliquots of the reaction mixture were pipetted into a microtiter plate, and absorbance was read at 340 nm for 5 min at 23°C with an ELISA plate reader (BioTek ELx808, Winooski, VT, USA). GST activity was measured in the mol of CDNB conjugate/ min/mg of protein.

#### 2.5.4 Catalase Activity

CAT assay was carried out with specifications, as mentioned previously, with minor changes [32]. We mixed 1.95 mL of phosphate buffer (50 mM, pH 7) and 1 mL of H_2_O_2_ solution with 0.05 mL of tissue homogenate (30 mM). At a wavelength of 240 nm, the absorbance of the final combination was measured. The catalase activity was calculated using the following formula:

CAT = δ OD ÷ E × Volume of a sample (mL) × protein (mg)

where δ OD is the change in absorbance per min, and E denotes the extinction coefficient of H_2_O_2_ at 240 nm (43.6M^-1^ cm^-1^). Protein levels were determined using the Lowery technique. Catalase activity was measured in µmol of H_2_O_2_/min/mg protein.

### 2.6 Blood Hyperlipidemia

The blood was collected from all experimental mice and centrifuged for 10 min at 3°C and 1400 rpm. 10 µl serum was added into 1 ml assay kit reagent and placed for 10 min at room temperature. The sample was placed in the Cholestech LDX Analyzer to analyze total cholesterol, TGL, HDL, LDL and random blood glucose.

### 2.7 Molecular Docking and MD Simulation

The tertiary structure of IL-1β, NRF2, and p-JNK was retrieved from the Protein Databank (PDB) (https://www.rcsb.org/) in PDB format using the PDB ID: 1ITB, 6QMK, and 3V6S [33]. The retrieved protein structure was subjected to preparatory procedures using the Dock prep module of UCSF Chimera v1.10.2 software program [34]. Notable procedures implemented during the preparatory steps included deleting heteroatoms, eliminating water molecules, and the cognate ligand. Furthermore, charge ions and missing hydrogen atoms were added. Subsequently, the resulting structure was taken for energy minimization using an energy minimization algorithm of the Swiss-PdbViewer v.4.10 software program [35]. The energy minimization was performed in vacuo with the GROMOS 43B1 parameter set and without field reaction. Noteworthy, the program automatically identifies missing side chains in protein residues and fixes them. Then, the affinity of Vit. B6 and their interaction with the active site residues of IL-1β, NRF2, and p-JNK were studied using molecular docking simulation using the VINA module in PyRx v 0.8 freeware [36]. An exhaustiveness of “6” was used during the docking simulation, and the complexes formed after docking were visualized using PyMOL v2.4.1 and LigPlot v2.2.4 for the 3D and 2D complexes, respectively [37]. Ultimately, the docking protocol was validated by redocking the co-crystallized ligand against the protein and superimposing it on the undocked co-crystallized ligand to obtain the root mean square of deviation (RMSD). Furthermore, we performed molecular simulations to validate docked complexes' structural stability using AMBER22 [38, 39]. The drug topologies were generated and processed for simulation using antechamber and parmchk2. Topology and coordinates files were used to minimize each complex in two stages: 1) the first round of minimization of 12000 steps and 2) the second round of minimization for 6000 steps was achieved. Each complex was sequentially heated and equilibrated for 50 ns. In the production stage, a 300 ns simulation for each complex was performed. The simulation was accelerated by using the GPU version of PMEMD.cuda and trajectories were processed by using PTRAJ and CPPTRAJ [40, 41].

### 2.8 Statistical Analysis

The bands of western blot results were scanned and assembled. Then, the statistical analysis of scan bands was analyzed for densitometry performed by using specialized computer-based software, *i.e*., ImageJ, followed by the use of GraphPad Prism5 to make histograms and graphs. One-way ANOVA followed by post hoc tests were performed to determine the significance statistically. The protein density was expressed in Arbitrary Units (A.U.s), and the mean ± S.E.M. # denotes a significant difference between the control group and cd-treated plus Vit. B6 treated mice, respectively: Significance ^*#^*P* < 0.05, ^**##^*P* < 0.01, ^***###^*P* < 0.001.

## 3 RESULTS

### 3.1 Vit. B_6_ Restored Neuronal Synapse and Memory Dysfunction in Adult Mice

**Fig. (1):**
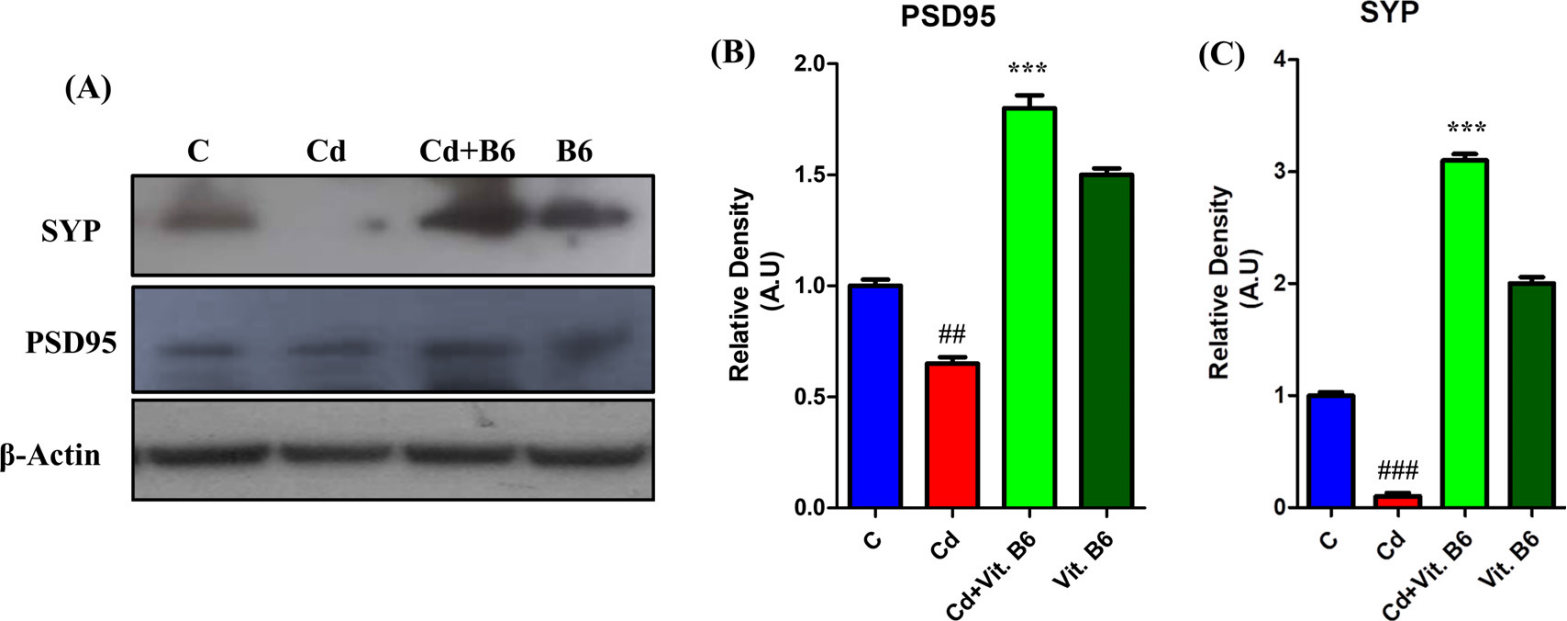
Vit. B6 enhanced pre- and post-synaptic protein expression against Cd in mice (**A**) The immunoblotting findings of synaptic protein Syp and PSD95 expression in the brain supernatant of experimental mice. The histograms display the relative densities in mean in AU ± SEM of PSD95 (**B**) and SYP (**C**). ##= *p* ≤ 0.01, ***= *p* ≤ 0.001 ### = *p* ≤ 0.001.

The western blot analysis revealed that Cd exposure led to a reduction in the protein expression of both pre-and post-synapse proteins, specifically Syp and PSD95 proteins (Fig. **1A**). In adult mice, the pre-synapse protein Syp was significantly inhibited by cadmium compared to the control group. However, in the group treated with a combination of Cd and Vit. B6 (Cd + Vit. B_6_), there was a notable increase in Syp protein expression compared to the Cd-treated group (Fig. **1B**). Similarly, the post-synapse protein PSD95 also decreased expression upon cadmium induction in contrast to the control group. Nonetheless, in the Cd+Vit. B_6_ group, the expression of Syp protein was significantly elevated compared to Cd alone (Fig. **1C**).

### 3.2 Vit. B6 Improved Memory in Adult Mice's Brain

**Fig. (2):**
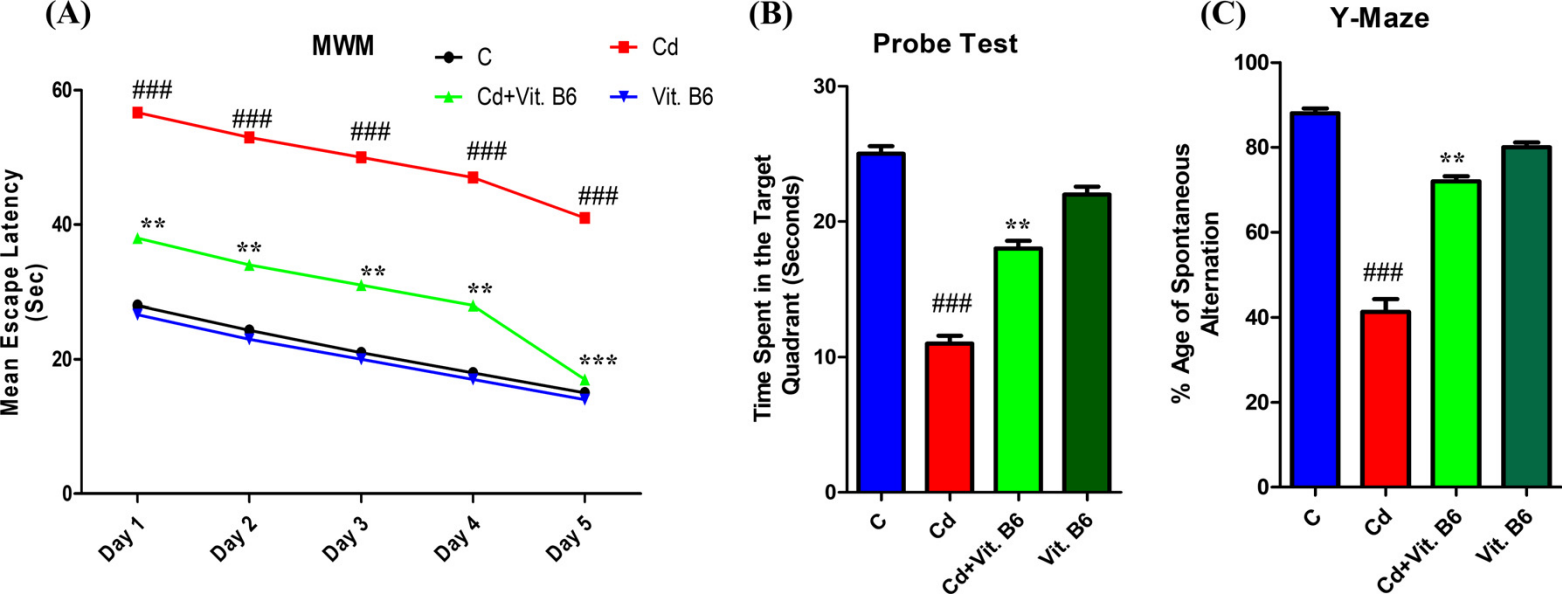
Vit B6 administration improved Cd-induced memory dysfunction in adult mice. Result of MWM (**A**), probe test (**B**) and Y-maze test (**C**) for all experimental groups. All the data represented as mean ± SEM., n=10. The significance of the comparison between control and Cd is denoted by the symbol “#,” whereas the symbol “*” is used to represent the comparison between Cd and Cd + Vit. B6. Significance: **p ≤ 0.01 and ***, ###*p* ≤ 0.001.

Among the experimental groups, the control mice displayed a decreasing mean escape latency from day 1 to day 5, indicating improved learning and memory performance (Fig. **2A**). In contrast, the Cd-treated mice exhibited a higher mean latency to reach the platform, suggesting impaired spatial learning. Remarkably, the combined treatment group (Cd+Vit.B6) showed a significant reduction in escape latency, while the mice treated with Vit. B6 (300 µg/kg) alone also demonstrated similar performance to control animals with reduced escape latency over the 5-day period. During the probe test, where the submerged platform was removed, the control mice spent more time in the platform quadrant, indicating better memory retention for the platform location. Similarly, the Vit. B6-treated mice also showed good memory after the control group. The only Cd-treated mice spent even less time in the quadrant, indicative of memory impairment. However, the combined treatment mice spent more time searching for the submerged platform in the quadrant, suggesting a potential improvement in memory function (Fig. **2B**). The measurement of latency and spontaneous alternation in the Y-maze is indicative of the mice’s memory performance. The control mice displayed a high level of spontaneous alternation, reflecting their superior memory capabilities, while the Cd-treated mice showed a decrease in spontaneous alternation compared to the control group. Intriguingly, the combined treatment mice exhibited an increase in spontaneous alternation as compared to the Cd-treated mice, indicating a potential enhancement of memory function. The mice treated with Vit. B6 also showed more spontaneous alternation compared to the combined group (Fig. **2C**).

### 3.3 Vit. B6 Inhibited NF-κb and Associated Neuroinflammation in Adult

**Fig. (3):**
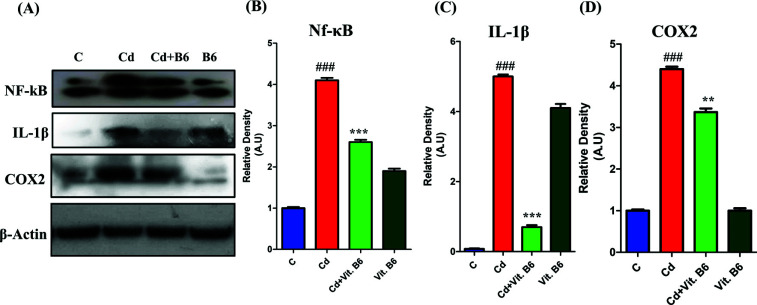
Vit. B6 inhibited NF-κB and COX2 proteins in mice. (**A**) Western blot analysis of NF-κB, IL-1β and COX2. The histograms illustrate the relative densities of NF-κB (**B**), IL-1β (**C**) and COX2 (**D**). The histogram demonstrates the average in A.U ± SEM. The significance of the comparison between control and Cd is denoted by the symbol “#,” whereas the symbol “*” is used to represent the comparison between Cd and Cd + Vit. B6. Significance**, *p* ≤ 0.01 and ***, ###*p* ≤ 0.001.

In the present study, we conducted a western blot analysis to assess the expression levels of NF-κB and IL-1β in mice from different experimental groups. As depicted in Fig. (**3A**), the Cd-induced mice exhibited significantly higher activation of NF-κB compared to the control group mice; however, in the group of mice treated with a combination of Cd and Vit. B6, a distinct result was observed, as Vit. B6 appeared to inhibit the activation of NF-κB (Fig. **3B**). Regarding IL-1β expression, the Cd-treated mice displayed elevated levels of IL-1β protein compared to the control mice. However, in the group of mice subjected to the combined treatment (Cd+Vit. B6), a different outcome was observed with Vit. B6 exhibiting inhibitory effects on IL-1β expression (Fig. **3C**). Furthermore, Cd induction resulted in the upregulation of cyclooxygenase 2 (COX-2), as evident from the Western blot analysis in Fig. **3D**. Notably, in the combined treatment group, Vit. B6 seemed to inhibit the expression of COX-2, leading to lower levels compared to the Cd-treated group. These findings suggest that Cd exposure induces the activation of NF-κB, elevation of IL-1β, and upregulation of COX-2, which may contribute to neuroinflammation and synaptic dysfunction. Interestingly, it should be noted that alone vit. B6 also has an anti-inflammatory effect as it reduces the expressions of neuroinflammatory protein markers compared to the Cd-alone treated group.

### 3.4 Vit. B6 Stimulated Nrf-2 to Inhibit p-JNK in Adult Mice Brain

**Fig. (4):**
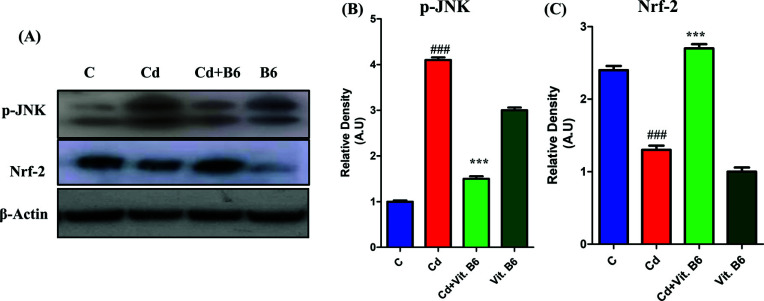
Cd-induced p-JNK and Vit. B6 stimulates Nrf-2 in adult mice brains. (**A**) Western blot results show that Vit. B6 decreases proinflammatory cytokines and increases anti-inflammatory cytokines in combined treated mice. Cd inhibits p-JNK in Cd-treated mice, as shown in (**B**), while Vit. B6 stimulates the expression of protein and stimulates Nrf-2, as shown in histogram (**C**). The significance of the comparison between control and Cd is denoted by the symbol “#,” whereas the symbol “*” is used to represent the comparison between Cd and Cd + Vit. B6. Significance: ***, ###*p* ≤ 0.001.

The western blot analysis reveals that the presence of Vit. B6 in mice treated with Cd leads to a noticeable decrease in the phosphorylation of JNK (Fig. **4A**). Specifically, the combined treatment group shows significant inhibition of p-JNK compared to the Cd-treated mice (Fig. **4B**). Moreover, our investigation also examined the effect of Vit. B6 on the hippocampal protein Nrf-2. The western blot analysis demonstrated that Vit. B6 treatment promoted Nrf-2 in Cd-treated mice compared to the control group. However, in the combined treatment group, the inhibition of Nrf-2 was observed in comparison to the Cd-treated mice (Fig. **4C**). These results suggest that Vit. B6 plays a role in modulating JNK phosphorylation, potentially offering neuroprotective effects against Cd-induced neurotoxicity. It is to be noted that only Vit. B6 group surprisingly induced the phosphorylation at JNK and reduced the expression of Nrf2 proteins. These findings are alarming and need to be resolved.

### 3.5 Effect of Vit. B6 against CD Cause Oxidative Stress

**Fig. (5):**
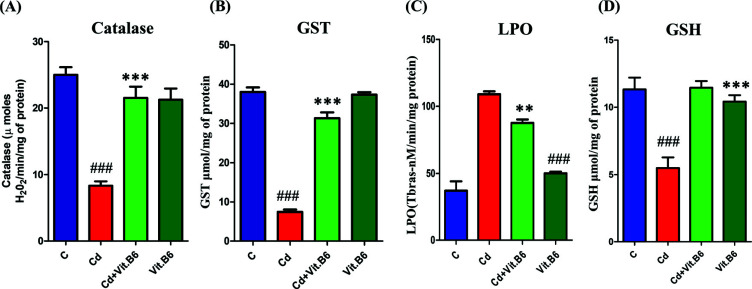
Vit. B6 reduced Cd-induced oxidative stress in adult mice brains. The results of antioxidant enzyme assays of (**A**) Catalase, (**B**) GST, (**C**) Lipid peroxidase (TBARS), and (**D**) Glutathione (GSH). These assays were conducted using brain homogenates from four groups of mice: control, Cd treated, Cd plus Vit. B6 treated, and Vit. B6 alone treated. The assays were conducted in triplicate and presented as the Mean ± SEM, n = 10. The significance of the comparison between control and Cd is denoted by the symbol “#,” whereas the symbol “*” is used to represent the comparison between Cd and Cd + Vit. B6. Significance: **p ≤ 0.01 and ***, ###*p* ≤ 0.001.

Within the cellular context, the presence of free radicals and the excessive generation of reactive oxygen species (ROS) beyond the cell's capacity is indicative of cellular exposure to cadmium (Cd). In this study, we investigated the effects of Cd on antioxidant defense systems, focusing on Catalase, GSH (Glutathione), and GST (Glutathione S-transferase) levels. Our findings revealed that Cd treatment led to a significant decrease in the activities of Catalase, GSH, and GST in the Cd-treated group (Figs. **5A**, **B**, **D**, Table **1**). However, in the combined treatment group (Cd+Vit. B6), there was a notable recovery observed in comparison to the Cd-treated group, indicating a potential protective role of Vit. B6 against Cd-induced antioxidant depletion. Interestingly, the group treated solely with Vit. B6 showed enhanced expression of antioxidant enzymes in all relevant groups, suggesting Vit. B6's positive impact on antioxidant defenses (Figs. **5A**, **B**, **D**). Furthermore, Cd exposure resulted in a significant increase in Lipid Peroxidation (LPO) levels in the Cd-treated group (Fig. **5C**). However, the combined treatment group demonstrated effective control over LPO levels, indicating a potentially ameliorating effect of Vit. B6 against Cd-induced oxidative damage. Taken together, our results suggest that Cd exposure induces oxidative stress, depleting antioxidant enzymes and increasing lipid peroxidation. However, the administration of Vit. B6 alongside Cd appears to counteract these effects, restoring antioxidant defense systems and attenuating lipid peroxidation. These findings highlight the potential of Vit. B6 as a therapeutic agent in mitigating Cd-induced oxidative damage in cells.

### 3.6 Vit. B6 Reduced Hyperlipidemia Induced by CD in Adult Mice

**Fig. (6):**
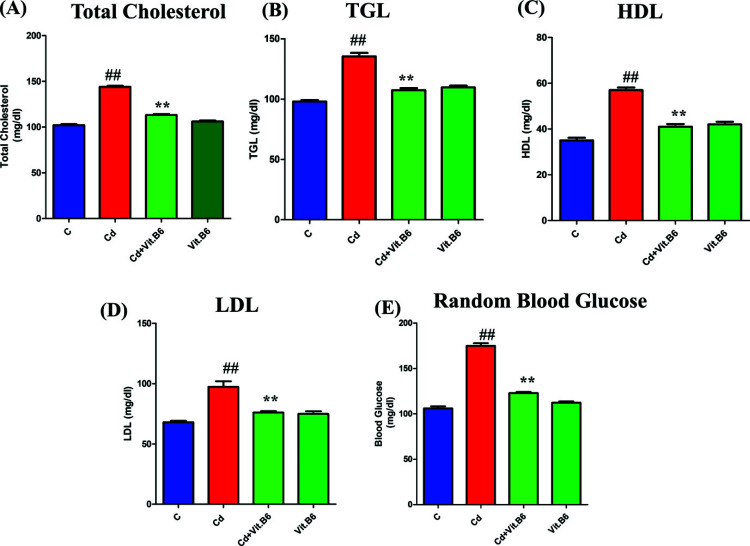
Vit. B6 reduced Cd-induced hyperlipidemia and sugar in blood in adult mice brains. The figure shows total cholesterol (**A**), TGL (**B**), HDL (**C**), LDL (**D**) and random blood sugar (**E**) levels in all four group mice: control, Cd treated, and Cd+Vit. B6 treated, and Vit. B6 alone treated (Mean ± SEM, n = 10). The significance of the comparison between control and Cd is denoted by the symbol “#,” whereas the symbol “*” is used to represent the comparison between Cd and Cd + Vit. B6. Significance: **, ##*p* ≤ 0.01

Hyperlipidemia is known to be a major contributing factor to diabetes (Mooradian, 2009). In our study, we analyzed the lipid profile of all experimental mice, including measurements of total cholesterol, TGL (Triglycerides), HDL (High-Density Lipoprotein), LDL (Low-Density Lipoprotein), and random blood glucose levels. The results demonstrated that Cd exposure led to the upregulation of total cholesterol, TGL, HDL, LDL, and random blood glucose levels, along with increased blood sugar levels (Figs. **6A**-**E**). On the other hand, the administration of Vit. B6 for 21 days on alternate days significantly reduced hyperlipidemia and blood sugar levels in the treated mice. These findings suggest that Vit. B6 may have a beneficial effect in mitigating the detrimental impact of Cd-induced hyperlipidemia and blood sugar elevation. The results suggest the potential of Vit. B6 as a therapeutic agent in managing hyperlipidemia and blood sugar levels associated with Cd exposure.

### 3.7 Vit. B6's Molecular Interactions Validate Anti-inflammatory Potential *via* Docking and Dynamics

**Fig. (7):**
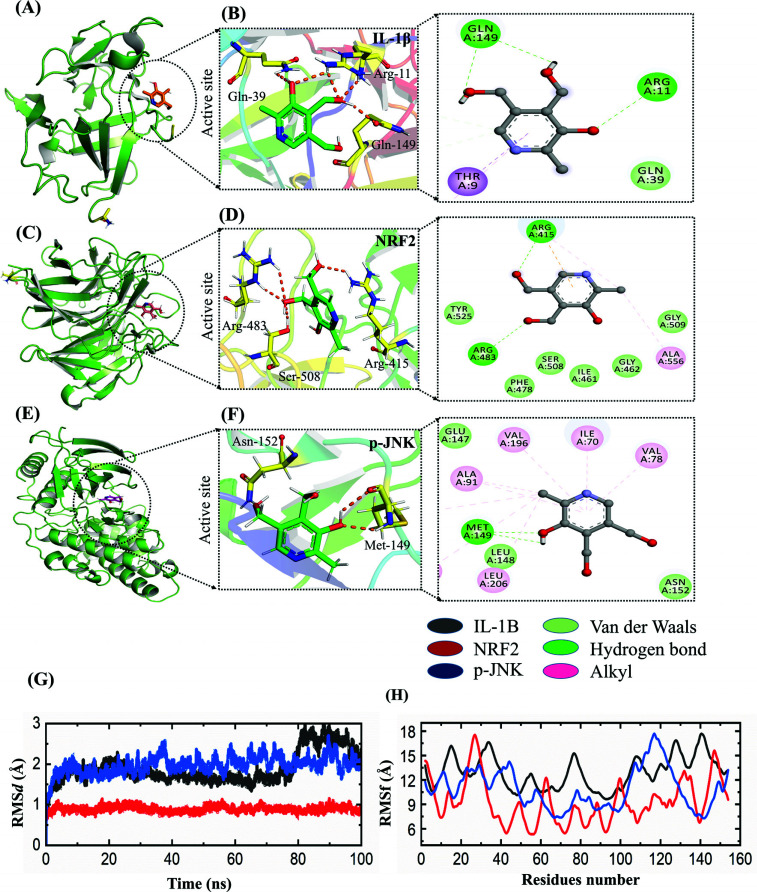
(**A-F**) Structural and binding analysis of IL-1β, Nrf2, and p-JNK with vit. B_6_. (**G**, **H**) Dynamic stability assessment and residues flexibility assessment of the IL-1β/vit. B_6_, Nrf2/vit. B_6_, and p-JNK/vit. B_6_ respectively.

This molecular docking exploration aimed to scrutinize the binding interactions between Vit. B6 and the target proteins IL-1β, Nrf2, and p-JNK, utilizing advanced computational techniques to uncover the binding site and the specific amino acid residues engaged in the vit. B6 interaction, providing insights into potential anti-inflammatory effects. IL-1β inhibition holds promise for treating diverse inflammatory disorders. IL-1β's docking score registered at -4.44 kcal/mol, firmly binding within the site and forming three hydrogen bonds with amino acids Gln39, Gln149, and Arg11 (Figs. **7A**, **B**). Similarly, Nrf2's docking score with Vit. B6 was -4.59 kcal/mol, forming four hydrogen bonds with Ser508, Arg483, and Arg415 (Figs. **7C**, **D**). Vit. B6's robust affinity to Nrf2 hints at activating Nrf2 signaling, bolstering cellular antioxidant defenses against oxidative stress-related diseases. For the p-JNK/Vit. B6 complex docking exhibited three hydrogen bonds with amino acids Asn152 and Met149 (Figs. **7E**, **F**). To validate docking predictions, molecular dynamics simulations gauged complex stability and residual flexibility. By analyzing root mean square deviation (RMSD) over time, the IL-1β/Vit. B6 complex showed initial stability up to 30 ns, reaching around 3.0 Å at 100 ns. Similarly, the Nrf2/Vit. B6 complex stabilized swiftly with minimal fluctuation over the last 100 ns. The p-JNK/Vit. B6 complex maintained steady RMSD levels, indicating stable dynamics with potential therapeutic impact (Fig. **7G**). Residue flexibility was assessed *via* root mean square fluctuation (RMSF) analysis (Fig. **7H**), showing consistent maintenance of JNK/vit. B6 at 9.0 Å, with minimal flexibility peaks. Across complexes, this stability hints at inhibitory potential by solidifying internal flexibility. Molecular Mechanics/Generalized Born Surface Area (MM/GBSA) analysis was conducted to evaluate binding free energy and deepen understanding of protein-ligand interactions. ΔG_bind values were calculated for the IL-1β/Vit. B6, Nrf2/vit. B6 complex, and p-JNK/vit. B6 complex, yielding -13.85 ± 2.05 kcal/mol, -11.35 ± 2.34 kcal/mol, -21.35 ± 2.12 kcal/mol, and -48.35 ± 2.63 kcal/ mol, respectively. These values suggest robust binding affinities. Overall, MM/GBSA analysis furnished insights into binding free energy and crucial molecular interactions, informing future drug design endeavors targeting the studied proteins.

## 4 DISCUSSION

In the current study, we examined the therapeutic potential of vit. B_6_ against Cd-induced oxidative stress, neuroinflammation, neural synapse alterations, and memory dysfunction in adult albino mice. Our finding collectively illuminates the significant impact of Vit. B6 in ameliorating the multifaceted detrimental effects of Cd exposure on the central nervous system. Notably, our results underscore the efficacy of Vit. B6 in countering Cd-induced oxidative stress within the brain of mice. The behavioral assessments, encompassing Y-maze, Morris water maze, and probe tests, unveiled a distinct memory impairment in Cd-treated mice, evident through higher escape latency and prolonged search for the platform. In contrast, Vit. B6 administration effectively restored memory function in the combined treatment group. This cognitive enhancement was paralleled by the inhibition of antioxidant enzymes by Vit. B6, thus effectively alleviating the oxidative stress provoked by Cd exposure [42, 43]. The observed parallels between our findings and previous studies showcasing the potential of Vit B6, Vit E, and selenium in reducing oxidative stress in cerebral regions further affirm the neuroprotective capacity of these nutrients [44, 45]. Furthermore, our exploration of Vit. B6's anti-inflammatory effects against Cd-induced neuroinflammation yielded compelling results. Interestingly, in the current study alone, vit. B6 also has an anti-inflammatory effect as it reduces the expressions of neuroinflammatory protein markers compared to the Cd-alone treated group. Vit. B6 exhibited a robust capacity to attenuate the inflammatory mediators COX-2, NF-κB, and IL-1B within the brains of adult albino mice. Such anti-inflammatory effects resonate with previous research demonstrating similar outcomes for other B vitamins [46]. Delving deeper, we conducted a comprehensive assessment of neuronal synapses and memory through western blot analysis and behavioral tests. Remarkably, Vit. B6 administration increased the expression of pre- and post-synapse proteins (SYP and PSD95), indicating improved synaptic function. This favorable synaptic alteration was mirrored in the improved memory observed within the combined treatment group compared to Cd-treated mice. These outcomes parallel previous associations between Vit. B6 intake, heightened cognitive function, improved memory, and enhanced learning capabilities [47]. Additionally, the analysis of lipid profiles in experimental mice showed that Cd exposure led to hyperlipidemia and increased blood sugar levels. In contrast, Vit. B6 treatment effectively reduced hyperlipidemia and blood sugar induction, suggesting its potential in managing these metabolic abnormalities associated with Cd exposure [48, 49]. These metabolic changes, coupled with hippocampal impairments observed in Cd-treated mice, contribute to the disruption of memory, learning, and cognitive abilities [50]. The result of Cd- treated mice showed high cholesterol, TGL, HDL, LDL and random blood glucose compared to Vit. B_6_-treated mice [51]. Our findings resonate with investigations highlighting the adverse impacts of heavy metals on metabolic health and memory, particularly in relation to hyperphosphorylation and diabetes [52]. Vit. B6 has anti-inflammatory and antioxidant properties and a co-factor role with different pathways, underpinning its potential to address the intricate interplay of oxidative stress, inflammation, and metabolic dysfunction [24, 53]. Vit. B6 has therapeutic effects and may involve preconditioning mechanisms and the vitagene network in neuroprotection. Preconditioning enhances cellular resilience against stress, while the vitagene network maintains cellular homeostasis [54-56]. Vit. B6 administration counteracts oxidative stress, neuroinflammation, and engages preconditioning pathways. It can improve synaptic function, memory, and modulates inflammatory markers. Further exploration is needed to understand the interplay of these mechanisms in mitigating the neurotoxic effects of heavy metal exposure. Furthermore, to validate the binding site predictions from molecular docking, we utilized molecular dynamics simulations. The goal was to unveil both the stability and residual flexibility of protein-ligand complexes. Our exploration of binding stability, assessed by calculated RMSD profiles, revealed distinct behaviors across the complexes. The IL-1β/ vit. B6 and Nrf2/vit. B6 complexes initially exhibited stability followed by fluctuations, in contrast to the p-JNK/vit. B6 complex which maintained stability throughout simulation. This suggests potential for robust therapeutic effects within the p-JNK/vit. B6 complex. Moreover, our examination of residue flexibility through RMSF emphasized controlled flexibility's significance for ligand recognition and binding stability. The combined insights from this study offer a dynamic comprehension of protein-ligand interactions, pivotal for advancing rational drug design strategies and optimizing therapeutic approaches. Overall, our study demonstrates that Vit. B_6_ acts as a promising therapeutic agent against Cd-induced neurotoxicity. Its anti-inflammatory and antioxidant properties, along with its ability to improve synaptic function and memory, make it a potential candidate for mitigating the adverse effects of Cd exposure. Further research is warranted to elucidate the underlying mechanisms and explore the clinical implications of Vit. B_6_ in the context of neurological disorders associated with heavy metal exposure.

## CONCLUSION

In conclusion, our study underscores the association between Cd-induced oxidative stress, neuroinflammation, and memory dysfunction in mice, as validated by behavioral tests and western blot analysis. Vit. B6 emerges as a potential therapeutic option, demonstrating efficacy in mitigating Cd-induced oxidative stress and restoring memory function. Its neuroprotective effects extend to the restoration of both pre-and post-synaptic memory, along with enhancing synaptic function. Furthermore, Vit. B6 exhibits regulatory effects on hyperlipidemia and blood sugar levels induced by Cd exposure, suggesting its potential in managing associated metabolic imbalances. The readily available nature in the market, coupled with its natural presence in various sources, positions it as a promising and accessible option. Our comprehensive findings depict Vit. B6 as a versatile agent, offering antioxidant, memory-enhancing, and metabolic-regulating properties, thereby holding promise for interventions in neurodegenerative diseases. Further research and clinical investigations are warranted to fully explore Vit. B6's potential as a safe and effective treatment for neurological disorders.

## Figures and Tables

**Table 1:** Effect of different groups on antioxidant enzymes and lipid peroxidation.

**Groups (µM/min/mL).**	**GSH (µM/mg of Protein)**	**Catalase (µM H_2_O_2_/min/ mg of Protein)**	**GST (µM/mg of Protein)**	**LPO (nM/min/mg of Protein)**
1.	12.23 ± 2	25.5 ± 2	40.2 ± 2	48.3 ± 2
2.	7.56 ± 1.4^≠≠≠^	8.6 ± 1.8^≠≠≠^	9.3 ± 2^≠≠≠^	110.3 ± 3^≠≠≠^
3.	11.28 ± 1***	23.7 ± 1.9***	32.4 ± 2***	93.6 ± 1.2***
4.	10.43 ± 1.5	23.6 ± 2	38.9 ± 1.8	50.5 ± 1

Symbols ### and *** show a significant difference at *p* < 0.001. Data are shown as mean ± SEM (n = 5). **Abbreviations:** CAT, catalase, GSH, glutathione; GST, glutathione reduced and LPO, lipid peroxidation. The results of each mice group are indicated as Mean ± SEM. # indicates the significance of control *vs.* Cd.

## Data Availability

The data that support the findings of this study are available from the corresponding author, [MZ], upon reasonable request.

## LIST OF ABBREVIATIONS

AD: Alzheimer's Disease
Aβ: Amyloid-beta
Cd: Cadmium Chloride
COX-2: Cyclooxygenase 2
GSH: Glutathione
LPO: Lipid Peroxidation
ND: Neurodegenerative Disease
NRF2: Nuclear Factor Erythroid 2-related Factor 2
PD: Parkinson's Disease
PVDF: Polyvinylidene Difluoride
ROS: Reactive Oxygen Species
